# Discrimination of Naturally-Occurring 2-Arylbenzofurans as Cyclooxygenase-2 Inhibitors: Insights into the Binding Mode and Enzymatic Inhibitory Activity

**DOI:** 10.3390/biom10020176

**Published:** 2020-01-23

**Authors:** Ericsson Coy-Barrera

**Affiliations:** Bioorganic Chemistry Laboratory, Facultad de Ciencias Básicas y Aplicadas, Universidad Militar Nueva Granada, Cajicá 250247, Colombia; ericsson.coy@unimilitar.edu.co; Tel.: +57-1-6500000

**Keywords:** cyclooxygenase-2, 2-arylbenzofurans, enzymatic activity

## Abstract

2-arylbenzofuran-containing compounds are chemical entities that can be naturally produced by several organisms. A wide-range of activities is described for several compounds of this kind and they are, therefore, valuable moieties for a lead finding from nature. Although there are in-vitro data about the activity of 2-arylbenzofuran-related compounds against cyclooxygenase (COX) enzymes, the molecular level of these COX-inhibiting constituents had not been deeply explored. Thus, 58 2-arylbenzofurans were initially screened through molecular docking within the active site of nine COX-2 crystal structures. The resulting docking scores were statistically analyzed and good reproducibility and convergence were found to discriminate the best-docked compounds. Discriminated compounds exhibited the best performance in molecular dynamics simulations as well as the most-favorable binding energies and the lowest in-vitro IC_50_ values for COX-2 inhibition. A three-dimensional quantitative activity-structure relationship (3D-QSAR) was also demonstrated, which showed some crucial structural requirements for enhanced enzyme inhibition. Therefore, four hits are proposed as lead structures for the development of COX-2 inhibitors based on 2-arylbenzofurans in further studies.

## 1. Introduction

Cyclooxygenase, COX, (or Prostaglandin H2 synthase), is a well-known enzyme that catalyzes the rate-limiting step in the formation of prostanoids such as prostaglandins (PGs) and thromboxane A2 (TxA2). These products are considered as important mediators of fever, pain, and inflammation [1]. Three COX isozymes are known such as the constitutive form including COX-1 (which exists in several tissues), the inducible form, COX-2 (which is expressed throughout inflammation), and the COX-1 splice variant, COX-3 [1]. In recent decades, the fact for understanding the distinct functions of COX isozymes has encouraged many studies, including those dedicated to the search for anti-inflammatory agents. In this sense, it is well-recognized that selective COX-2 inhibitors have been developed as securer substitutes for the nonsteroidal anti-inflammatory drugs (NSAIDs), mainly due to the adverse effects caused by the inhibition of the COX-1 enzyme. Therefore, a pronounced interest for finding selective anti-COX-2 agents still remains [2].

Several types of compounds (natural or synthetic) with anti-COX-2 properties have been discovered in recent years. Several chemical entities have been studied as selective COX-2 inhibitors with interesting and important potential [3]. However, the most important ones are those named coxibs (such as celecoxib, rofecoxib, and valdecoxib), which have been approved due to the lower adverse effects in comparison to the traditional NSAIDS (non-steroidal anti-inflammatory drugs) [3]. However, it has recently been reported that some coxibs and other selective COX-2 inhibitors cause non-favorable gastrointestinal and cardiovascular events [3,4]. Because of that, novel agents for COX-2 inhibition are still required.

A huge interest on benzofuran-containing compounds has been originated several years ago because of their important biological and pharmacological properties and their extensive natural occurrence [5]. Benzofuran moiety is considered to be an indole isostere (a potent pharmacodynamic moiety exhibiting anti-inflammatory activity) and, consequently, several anti-inflammatory benzofuran-based compounds have been studied [6,7]. However, despite the plethora of reported benzofuran derivatives, 2-arylbenzofurans have not been sufficiently explored at a molecular level for COX-2 inhibition in spite of some of them exhibiting selective in-vitro anti-COX-2 activity [8,9]. Thus, as part of the research on anti-inflammatory compounds of natural origin, a combined in-silico study comprising different computational approaches/tools were used on the basis of three motivations: (1) discriminating putative COX-2 binders through a molecular docking-based screening of 2-arylbenzofuran-related compounds, within the active site of nine reported crystal structures of COX-2, (2) describing some insights into the binding mode of the best-docked compounds using molecular dynamics and binding energy calculations, and (3) recognizing some structural requirements of the best-docked compounds toward COX-2 inhibition by means of comparative molecular field analysis (CoMFA). This last purpose required the expansion of the in-silico predictions to experimental results through the in-vitro COX-2 inhibitory assay performed on an in-house collection of 2-arylbenzofurans. Results revealed four very important hits to be used in future studies for developing novel selective COX-2 inhibitors based on 2-arylbenzofurans.

## 2. Materials and Methods 

### 2.1. Protein Preparation

The X-ray crystallographic structure of nine COX-2 proteins (from *Mus musculus*) were obtained from the Protein Data Bank (PDB) (http://www.rcsb.org) at a resolution < 3.00 Å (Table 1). Water molecules, ligands, and other heteroatoms were removed from the protein molecules. The addition of hydrogen atoms to the proteins was then performed. These crystallographic structures were retained without any processing for molecular docking. The co-crystallized inhibitors/substrates were employed to define the corresponding active site, the flexible residues within this active site, and a validation criterion of docking calculations (re-docking). By means of a literature survey, a maximum of five common residues involved in the binding site were defined as flexible residues: Arg106, Val335, Ser339, Tyr341, and Ser530. PDB files of proteins were effectively prepared using the AutoDock/Vina (Molecular Graphics Lab, The Scripps Research Institute, La Jolla, CA, USA) plugin under PyMOL 2.3 (Schrödinger LLC, New York, NY, USA), and saved as pdbqt files. COX-2 structures were also superimposed by sequence alignment for calculating the root-mean-square deviation (RMSD) and sequence similarity using Discovery Studio Client 16.1 (Biovia, San Diego, CA, USA).

### 2.2. Ligand Preparation 

A targeted, custom-made library comprising only 2-arylbenzofurans (*n* = 58) was compiled from the information of an in-house collection of isolated compounds as well as different published works reporting anti-inflammatory activity of structurally-related 2-arylbenzofurans of natural origin. Compiled compounds were three-dimensionally (3D)-sketched using the molecular builder and visualization tool Avogadro 1.2.0 (open-source, http://avogadro.cc). A Monte-Carlo randomized conformational search was then performed, without any geometrical restrictions, using the semi-empirical AM1 parametrization implemented in the Spartan’14 software (Wavefunction, Irvine, CA, USA) with a limit of 500 conformers. Energetically-lowest stable conformers, within a 6 kcal/mol energy range, were geometrically optimized at a density functional theory (DFT) level using B3LYP hybrid functional and 6–311++G (2df, 2p) basis set that is also implemented in Spartan’14. After structural optimization, harmonic vibrational frequencies were calculated at the same level of theory to verify the reliability of the stationary point at a minimum and all structures converged successfully. During geometry optimization, the highest energy occupied molecular orbital (HOMO) and lowest energy unoccupied molecular orbital (LUMO) were also computed for further analysis. The verified, optimized ligand structures were saved as PDB-files for further molecular docking and molecular dynamics analysis. Additionally, structural similarity analysis of 2-arylbenzofurans was performed using the substructure fragment dictionary-based binary fingerprint descriptor (FragFp) implemented in the open-source data visualization and analysis program DataWarrior [17]. The absorption, distribution, metabolism, and excretion–toxicity (ADMET) properties of test compounds were computationally predicted using the Free ADMET Filtering tool (FAF-Drugs4, http://fafdrugs4.mti.univ-paris-diderot.fr).

### 2.3. Molecular Docking

**Figure A1:**
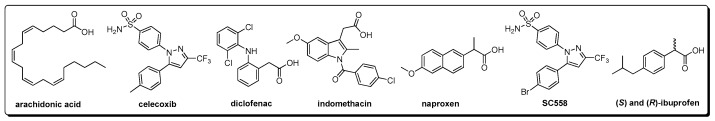
Structures of natural ligand and selective and non-selective COX-2 inhibitors.

Molecular docking calculations were performed by means of Autodock/Vina (Molecular Graphics Lab, The Scripps Research Institute, La Jolla, CA, USA) using PyMOL 2.3 (Schrödinger LLC, New York, NY, USA) and running on a Ubuntu 12.04 server equipped with a dual Intel Xeon^®^ processor CPU @ 2.6 GHz (32 CPU), 64 GB DDR3 RAM. Prepared proteins and ligands were used for simulations using flexible residues. The selection of such flexible residues within the active site was based on a position at 4.0 Å from co-crystallized ligand (i.e., substrate or inhibitor, as presented in Table 1 and Figure A1). Thus, the active site was placed into a 24 × 24 × 24 Å cubic box at the geometrical center of the set of selected flexible residues, which has 0.375 Å as grid point spacing. Ten different pdbqt-files of each ligand were used as replicates to be docked into the flexible active site of prepared enzymes to evaluate convergence. Co-crystallized ligands were also docked in order to assess the docking performance through a re-docking strategy. Additionally, the specificity and sensitivity of the docking protocol, 15 compounds of diverse chemical nature, which have IC_50_ < 4 μM, against COX-2 (3LN1) were compiled from ChEMBL [18]. Furthermore, 50 decoys per each active compound were compiled from the directory of useful decoys—enhanced (DUD-E) [19]. Thus, decoys and bioactive compounds were processed using the same docking protocol. The resulting data was then assessed through a receiver operating characteristic (ROC) and scores enrichment using the screening explorer webserver [20]. Once the docking parameters were validated, the docking simulations of test 2-arylbenzofurans were performed. Docking results were initially examined in PyMOL 2.3 and the resulting poses were top-ranked regarding the Vina scores (kcal/mol). Molegro Virtual Docker 6.0 (CLC Bio Company, Aarhus, Denmark) was secondly used to evaluate the best-poses of best-docked compounds as a rescoring strategy. RMSD of atomic positions of the best poses between replicates was calculated through an in-house bash script. Discovery Studio Client 16.1 (Biovia, San Diego, CA, USA) and Maestro 11.8 (Schrödinger, Cambridge, MA, USA) were used to visualize the respective 3D models and 2D residual interactions diagrams, respectively, using the pdbqt outputs for the highest-scored ligands.

### 2.4. Statistical Analysis

Descriptive and inferential statistics tests were carried out using R project software version 3.0.2 (R Foundation, Vienna, Austria). Principal component analysis (PCA) was also carried out in SIMCA 14.0 software (Umetrics, Umeå, Sweden) using the docking scores in order to observe plausible relationships with structural variations among test 2-arylbenzofurans.

### 2.5. Molecular Dynamics Simulations

The ligand’s best poses from selected docked (ligands) and crystal (COX-2, 3LN1) structures were chosen as inputs for molecular dynamics (MD) simulations of ligand-enzyme complexes and the apoenzyme. Such simulations were carried out on the systems comprised of COX-2+celecoxib, COX-2 + **11**, COX-2 + **29**, COX-2 + **42**, and COX-2 + **49** (obtained after molecular docking). These systems were individually subjected to an additional 150-ns conventional MD simulation for exploring the performance over the time of these ligands within the active site of the enzyme. These MD simulations were run in Gromacs 5.0.5 (open source, http://www.gromacs.org) on an Ubuntu 12.04 server, using NPT (constant pressure and temperature) and periodic boundary conditions, as previously reported [21]. Hence, docked ligands were prepared by adding hydrogen atoms in UCSF Chimera (UCSF, CA, USA) and the resulting pdb-file was uploaded to the atb server (http://compbio.biosci.uq.edu.au/atb/) to add the respective Gromo53a6 force field and get the itp-type topology file. COX-2 topologies were obtained in Gromacs using the Gromos53a6 force fields, due to the presence of the Heme group. The simple point charge (SPC) water model was then implemented for solvation in a triclinic box using a 1.0-nm margin distance. 0.10 M NaCl was added to the simulation systems and water molecules were randomly replaced until neutrality. An energy minimization through a 2000-steps steepest descent method was then used. NVT (constant volume and temperature) equilibration at 310 K during 500 ps, followed by NPT equilibration during 1000 ps using the Parrinello-Rahman method at 1 bar as a reference were done on the systems using position restraints. Lastly, solute position restraints were released and a production run for 90 ns was performed. Temperature and pressure were kept constant at 310 K and 1 bar, respectively. Coordinates were recorded in a 1 fs time step. Electrostatic forces were calculated using the particle-mesh Ewald (PME) method. Periodic boundary conditions were used in all simulations and covalent bond lengths were constrained by the LINear Constraint Solver (LINCS) algorithm.

### 2.6. Binding Free Energy Analyses

Binding-free energy was calculated using the g_mmpbsa tool [22] (open source drug discovery consortium (OSDD), New Dehli, India). This tool calculated components of the free energy of the protein–substrate binding (ΔG_Bind_) using the molecular mechanic/poisson-boltzmann surface area (MM/PBSA) method [23,24]. In this method, ΔG_bind_ calculation between a protein and a ligand is carried out by ∆G_bind_ = ∆H − T∆S ≈ ∆E_MM_ + ∆G_sol_ − T∆S, ∆E_MM_ = ∆E_internal_ + ∆E_electrostatic_ − ∆E_vdW_, ∆G_solv_ = ∆G^elec^_solv_ + DG^vdW^_solv_, where the total gas phase energy on the binding of MM energy is ΔE_MM_, the free energy of solvation is ΔG_solv_, and the entropy contribution is TΔS. A Poisson-Boltzmann model was used to compute the electrostatic solvation energy in a continuum solvent. The derivation of a non-polar solvation energy term was computed as a solvent-accessible surface area (SASA). ΔE_MM_ were calculated using the Lennard-Jones and Coulomb potential [23,24]. ΔG_Bind_ was used to analyze the binding associations between COX-2 and selected ligands (i.e., celecoxib, 11, 29, 42, and 49) by decomposing the total binding-free energy into each residue. The binding energy calculations of the selected ligands were performed for 100 snapshots taken at an interval of 500 ps during the last stable 40-ns MD simulations.

### 2.7. COX-2 Inhibition Assay

The ability of selected compounds to inhibit bovine COX-2 was determined using an enzyme immunoassay (EIA) kit (Cayman Chemical Company, Ann Arbor, MI, USA), according to the reported method [8]. The activity was expressed as half-maximal inhibitory concentrations, IC_50_ (in μM), obtained after non-linear regression, using the program GraphPad Prism version 5.00 (GraphPad Software, San Diego, CA, USA).

### 2.8. Comparative Molecular Field Analysis (CoMFA)

The best-docked poses of those test 2-arylbenzofurans experimentally evaluated against COX-2 were merged and aligned by means of particular tethers placed on benzofuran and aromatic rings moieties, using the molecular overlay tool included in the software Discovery Studio Client 16.1 (Biovia, San Diego, CA, USA). Compound **11** was selected as a template to select tethers. The aligned molecules set (in an sdf-file) was randomly divided into two subsets (training and test sets, corresponding to 70% and 30%, respectively). Comparative molecular field analysis (CoMFA) analysis was then performed using Open3DQSAR, using the standard protocol [25]. The experimental in-vitro COX-2 inhibition (expressed as IC_50_ in M) was converted into a negative logarithmic form (*p*IC_50_) and then used as an independent variable. The models were validated by leave-one-out (LOO) and leave-many-out (LMO) methods. The quality of the models after validation was evaluated on predicting the independent variable for the test set.

## 3. Results and Discussion

### 3.1. 2-Arylbenzofuran-Related Chemical Space

**Figure 1:**
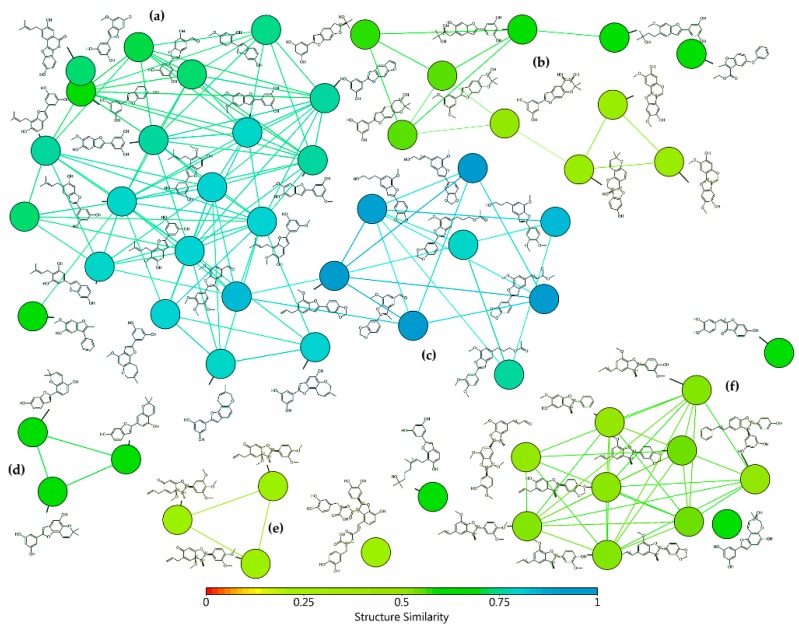
Similarity chart of 2-arylbenzofurans 1–58 after structure similarity analysis using the substructure fragment dictionary-based binary fingerprint descriptor (FragFp) ([17]. Colors according to the heatmap based on the structure similarity index between 0 (red) and 1 (light blue). Clusters according to: (**a**) moracin-type, (**b**) 2*H*-pyran- and benzofuro[6.5-*b*]furan-containing, (**c**) propyl(en)-susbtituted, (**d**) dihydropyran-containing, (**e**) furocyclohexadienone-type, and (**f**) licarin-type.

**Figure A2:**
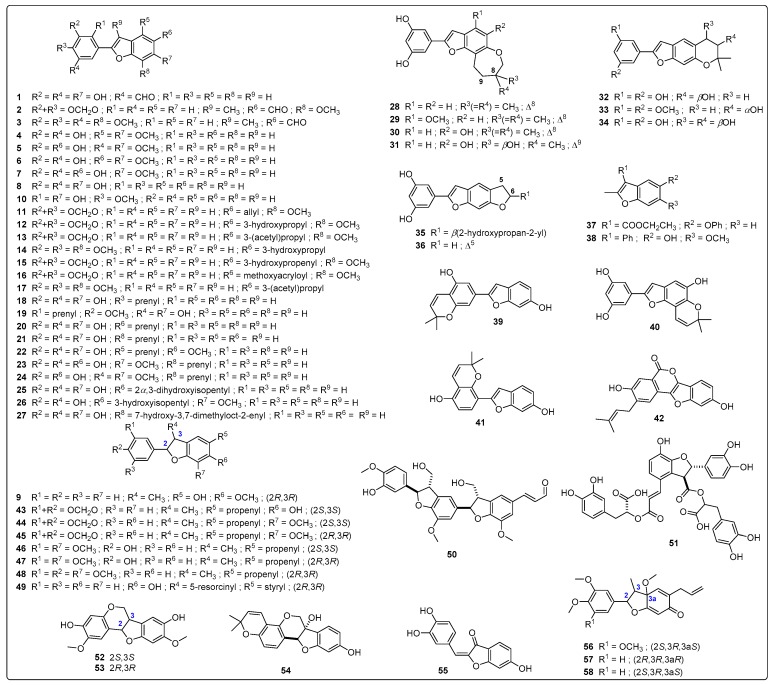
Structures of the test 2-arylbenzofuran-related compounds.

The selection of test 2-arylbenzofuran-type compounds was based on three parameters: (1) natural origin, (2) enlarging the chemical space of an in-house collection (*n* = 26) of 2-arylbenzofurans, and (3) having an anti-inflammatory effect (preferably, inhibition of enzymatic activity or expression of COX-2). Hence, a targeted, custom-made library, comprising 58 2-arylbenzofurans, was then compiled. Common names of these compounds (**1**–**58**) are found in Table A1 and their structures are presented in Figure A2. The 2-arylbenzofuran-related chemical space was examined to characterize it and facilitate the data analysis according to structural fragments. Therefore, a structural similarity analysis between test compounds was performed using the FragFp descriptor available in DataWarrior software [17]. Compound **11** was selected as a reference to compute the similarity index due to its good COX-2 inhibitory activity, as previously reported [8]. The resulting similarity chart is presented in Figure 1.

After such an analysis, some clusters were shown, which involved similarity FragFp indexes between 0.93 and 0.28, according to the heatmap based on the structure similarity index between 0 (red) and 1 (light blue) (Figure 1). Clusters were related to different classes of substituted benzofurans, which were then subdivided into moracin-type (including isopentyl-substituted and dihydrooxepine-containing) (FragFp 0.93–0.69), 2*H*-pyran- and benzofuro[6.5-*b*]furan-containing (FragFp 0.67–0.38), propyl(en)-susbtituted (FragFp 0.90–0.71), dihydropyran-containing (FragFp 0.66–0.62), furocyclohexadienone-type (FragFp 0.28), licarin-type (i.e., 2-aryldihydrobenzofuran) (FragFp 0.50–0.46), and other four ungrouped compounds (FragFp 0.67–0.59). This structure similarity analysis clustered quantitatively the test compounds into six classes, which indicated these compounds have particular moieties/fragments to have further analysis.

### 3.2. Molecular Docking Simulations

**Figure 2:**
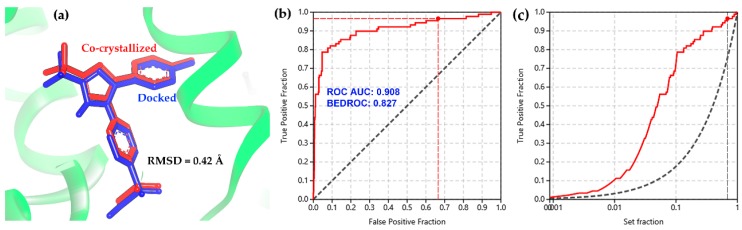
(**a**) Docked (blue sticks) and co-crystallized (red sticks) superposition of celecoxib after re-docking within the active site of COX-2. (**b**) Receiver operating characteristic (ROC) and (**c**) enrichment curve for the benchmarking of the docking protocol using a set of active compounds and decoys against COX-2 (3LN1).

Molecular docking was chosen as a first-line discrimination of bioactives from the above-mentioned chemical space related to 2-arylbenzofuran compounds due to the capacity to simulate the binding of small compounds within the active site of enzymes, as a structure-based discrimination procedure for the in-silico prediction of putative competitors [26,27,28]. In this regard, some concerns should be considered using accurate parameters for suitable selection of sampling and scoring procedures during structure-based screening [29,30]. Therefore, the molecular docking of the co-crystallized ligands for each enzyme structure (ES1–ES9, Table 1) indicated a good performance of the present docking protocol since these re-docking calculations resulted into low conformational RMSD values (<0.5 Å), such as the docked/co-crystallized superposition of celecoxib presented in Figure 2a. Furthermore, the docking performance was also evaluated through a benchmarking approach from docking results of 15 known selective COX-2 inhibitors in comparison to the binding scores of a group of decoys (*n* = 750). Calculation of the area under the curve (AUC) from ROC curves was used to estimate the sensitivity and specificity of the docking protocol [31]. AUC and the Boltzmann-enhanced discrimination of the receiver operating characteristic (BEDROC) resulted in 0.908 and 0.827, respectively. Therefore, the validation of the docking protocol was considered successful (Figure 2b). This evaluation reached true positive identification over 92% of the active compounds involving a recognition within 10% of the test compounds (Figure 2c).

**Figure 3:**
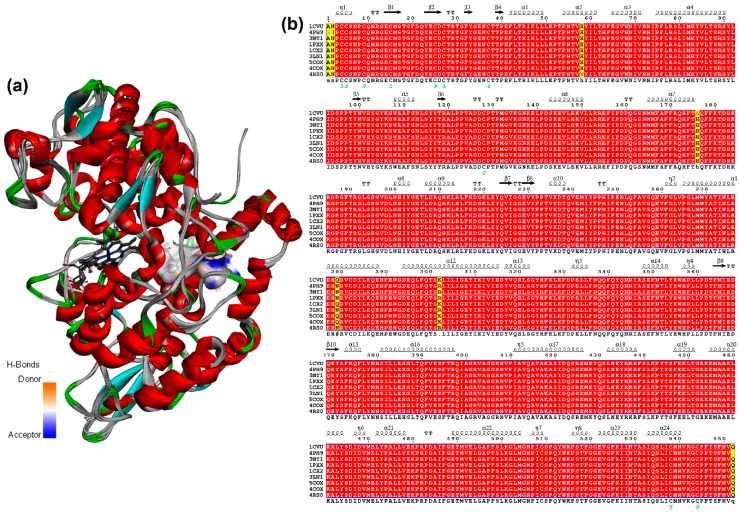
(**a**) Superposition of chains-A from nine COX-2 structures (represented as solid ribbons) after sequence alignment. The active site is highlighted by the *H*-bond donor/acceptor surface, based on the heatmap (orange = donor, blue = acceptor). The HEME group as grey sticks. (**b**) Sequence alignment chains-A from 9 COX-2 crystal enzymes. The figure was produced using ESPript (http://espript.ibcp.fr).

Once the parameters were adequately validated, each geometrically-optimized structure **1**–**58** was docked into the active site of each COX-2 structures (ES1–ES9, Table 1) using AutoDock/Vina. These enzyme structures ES1–ES9 were retrieved from the PDB and their respective data are shown in Table 1. These *M. musculus*-derived COX-2 structures were selected to compare the performance of selective (S) and non-selective (NS) COX-2 inhibitors and 2-arylbenzofurans, as a discriminating initiative using the re-docking strategy, to assess the predictive potential of the simulation protocol. It was, therefore, required to retrieve COX-2 structures bound to common anti-inflammatory drugs (i.e., S/NS COX-2 inhibitors) and the natural ligand. In addition, in spite of the importance to include human COX-2, they were not used in the present docking study. Other *Mm*COX-2 PBD structures were discarded due to some issues related to sequence, residues, and a bound ligand. Such facts were taken as criterion to select the test COX-2 structures. Furthermore, the active site was adopted according to the information described in the referenced studies. After a detailed scrutiny of such information, five common residues involved in the binding site were initially defined (i.e., Arg106, Val335, Ser339, Tyr341, and Ser530). They were then used in the present docking study as flexible residues as well as those located at 4.0 Å from the co-crystallized ligand. These structures used for docking consist of very similar COX-2 protein sequences in a different solution when the enzyme was co-crystallized with a ligand or unbounded. These structures ES1–ES9 presented small alterations on the tertiary structure depending on the crystallization protocol. In addition, the presence of the inhibitor bound to the active site can also induce changes in the 3D-conformation of the binding site and modify the accessibility of some residues for ligand interaction. These facts were examined on superimposing the 9 chain-A 3D-structures of COX-2 after sequence alignment (Figure 3), which exhibited slight differences in a primary and tertiary structure (i.e., sequence similarity 99.2%–99.8% and RMSD 0.35–0.50 Å (Figure 3a), respectively). The differences between these COX-2 sequences were found to be related to particular residues such as Asn34, Ala33, His90, His208, Gln310, and Lys333 (Figure 3b), but the active site remained identical. Therefore, the same ligands docked into the same binding site of different crystal structures of the same enzyme is very important to statistically support the docking results.

#### 3.2.1. Performance and Trends of the Docking Results

Docking results were examined by descriptive statistics to explore the trends and performance of the docking protocol, on one hand, and visualize and rationally select the best-docked compounds to discriminate putative binders of COX-2 on the other. After 10 replicates per calculation of benzofuran-related compounds **1**–**58** with nine crystal structures of COX-2 (ES1–ES9), the docking results were expressed as Vina Scores (in kcal/mol) and the best pose into the active site of each enzyme structure was also achieved. The mean docking scores, the standard deviation (SD) among replicates, and the RMSD (in Å) of atomic positions between the best poses after 10 replicates for each test benzofuran docked with each COX-2 structure are listed in Table A2. In general, the SD values were found to be within the 0.02–0.55 range, which indicates good reproducibility for docking calculations. However, the RMSD values exhibit high variability (i.e., 0.049–1.582 Å range). However, the most RMSD values were below 0.8 Å, which demonstrated good convergence in the calculated complexes formation. As expected, arachidonic acid (AA) exhibited the highest variability due to the long chain-derived conformations (14 rotatable bonds), but planar (more rigid) structures exhibited the lowest SD and RMSD values. 2-arylbenzofurans possessing an additional ring (e.g., pyran, furan, or oxepine) exhibited overall stronger docking scores (<−10 kj/mol). The previously mentioned facts were confirmed with the global mean Vina scores (GMVS) for the docking results with all COX-2 structures (Table 2).

Additionally, docking scores were found to be adequately distributed and reproducible. The relative standard deviation (RSD) percentages resulted in the 2.7%–33.2% range, but only 16.3% of the RSD values were above 10% and 3.6% of the RSD values above 20%. These facts are a good indication of the reproducibility. It is well-recognized that the docking procedure has a limitation related to distinguishing the correct pose among the generated scoring positions. However, the difference in the best poses among replicates were considered to be convergent since 76.3% of the RMSD values were below 0.6 Å. A similar trend was observed with inhibitors and the respective co-crystallized structure after re-docking calculations (RMSD < 0.5 Å).

**Figure 4:**
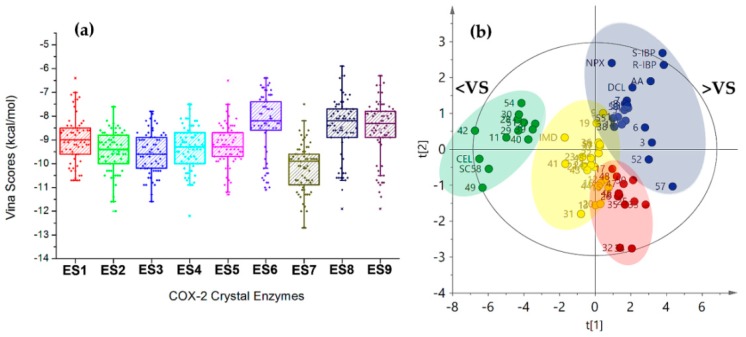
(**a**) Boxplot from Vina scores after docking between 2-arylbenzofuran-related compounds and nine crystal structures of COX-2. (**b**) Score plot from Principal Components Analysis (PCA) on Vina scores after docking of test compounds with COX-2 structures. Clustering: green (group 1), dark blue (group 2), Yellow (group 3), and red (Group 4). <VS = lowest Vina Scores. >VS = Highest Vina Scores.

Regarding the behavior of Vina scores among COX-2 structures (ES1–ES9), a similar distribution was found as shown in the respective boxplot (Figure 4a), which was built from the resulting mean docking scores. Mean values per COX-2 structure were similar for ES1–ES5, as a first group, and ES6, ES8, and ES9 as a second one. ES7 exhibited the lowest mean Vina score among enzymes and the lowest docking score (−12.7 ± 0.2 kcal/mol) corresponding to celecoxib (cel). These facts mean that the particular behavior of the ligands into each binding pocket was slightly different, which can be understood as a consequence of structural adaptations of the active site by the presence of the co-crystallized ligands. This issue should be considered a very important factor in the moment to get more reliable results during docking studies.

Trends within docking results were also inspected by multivariate statistics in order to identify the plausible relationship between docking scores and structural variations of 2-arylbenzofurans. A PCA was performed using the Vina scores dataset. The first two components explained the 82.3% of the total variance with good model fitting (R^2^ = 0.823 and Q^2^ = 0.778 for the principal component 1 (PC1) and PC2). The resulting score plot (PC1 vs. PC2, Figure 4b) demonstrated good differentiation of the compounds by variance according to the Vina scores along COX-2 structures. Hence, this unsupervised analysis clustered the compounds in four groups, which is shown by discriminating across PC1 depending on mean Vina scores (66.2% explained variance). Group 1 (green) consisted of the best-docking 2-arylbenzofurans (such as **11**, **28**, **29**, **30**, **40**, **42**, **49**, **51**, and **54**) with a comparable profile to the selective COX-2 inhibitors celecoxib (cel) and SC558. 2-arylbenzofurans possessing an additional cycle (such as pyran, furan, and oxepine) were the predominant structural feature in group 1. In contrast, group 2 (dark blue) clustered the weakest-docking 2-arylbenzofurans with profiles related to those of arachidonic acid (AA), indomethacin, and naproxen. This group was characterized by unsubstituted moracin-type compounds as well as some dihydrobenzofurans. Groups 3–4 (yellow and red) exhibited their separation by dual incidence of PC1 and PC2, whose separation along PC2 was mainly dependent by the scoring behavior with ES6 and ES8. These results indicated that the PCA led to discriminated bioactive compounds, according to the molecular docking performance.

#### 3.2.2. Selection of the Best-Docked Compounds

An examination of the previously mentioned docking results for compounds **1**–**58** (Table 2) was performed to select the best-docked compounds as putative COX-2 binders. Hence, from the global mean Vina scores (GMVS < −10.5 kj/mol)), best-pose convergence (RMSD < 0.5) and scoring reproducibility (%RSD <6%) along nine COX-2 structures, the compounds 9′-*nor*-7, 8-dehydro-isolicarin-B (**11**), moracin H (**29**) psoralidin (**42**), and maximol (**49**) were established as the best-docked compounds among the test set. These selected compounds were, therefore, clustered in the green group of the PCA-derived score plot (Figure 4b), which exhibits the lowest-scoring behavior. Other compounds in this group were not selected because they did not meet completely the previously mentioned features (i.e., GMVS, RMSD, and %RSD). In addition, Molegro Virtual Docker (MVD) was also used to simulate the intermolecular interaction as a rescoring strategy. An identical trend was obtained, so the best-docked compounds used in Vina exhibited the best MolDock scores (i.e., cel = −163.2, **49** = −156.9, **42** = −156.6, **11** = −139.1, **29** = −136.2), which is another indication of the good performance of the docking protocol and the filtering by descriptive and multivariate statistics. These four compounds were used for the first time to simulate their interaction within the active site of COX-2 using a validated docking protocol. However, compound **11** was previously evaluated against COX-2 enzyme (affording an IC_50_ of 3.32 μM) [8] and compound **42** was reported to exhibit good ability to reduce the COX-2 expression at 50 µM [32].

#### 3.2.3. Binding Mode and Residual Interactions of the Best-Docked Compounds

**Figure 5:**
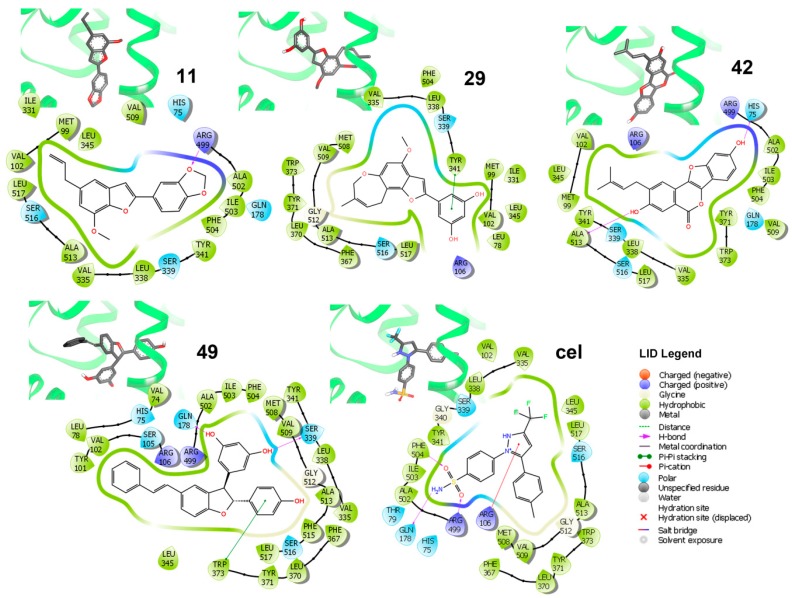
Three-dimensional (3D) models and ligand interaction diagrams (LID) of COX-2·ligand complexes of the lowest-scored pose of the best docked compounds **11**, **29**, **42**, and **49**, as well as celecoxib (cel). In LID, light green, aquamarine, and purple lines depict the active site surface. Dark green arrows indicate the π-π stacking between residues and aromatic moieties of compounds. Magenta arrows indicate the *H*-bonding. Residues are differentiated by colors according to the interaction type as indicated in the LID legend: hydrophobic (green), polar (aquamarine), and a positive charge (purple). 3D-models showed docked compounds in bold sticks and enzymes as a light green cartoon.

Binding modes of the best-docked 2-arylbenzofurans with the COX-2 structure ES7 (PDB code: 3LN1) was examined looking for important/crucial interactions between active site residues and ligand moieties, according to the individual 2D-residual interaction diagrams (Figure 5).

Analysis of these diagrams was useful to delineate some insights into the binding mode of the simulated complexes, which showed some important hydrophobic and polar interactions. These interactions were taken as key contacts and an indication of the importance of the presence of the hydroxiaryl moiety for interacting with the respective active site residues to stabilize the simulated enzyme-ligand complexes. Particularly, celecoxib exhibited *H*-bonds between Phe504, Arg499 (as donors), and Gln178 (as acceptor) and sulfonamide as well as Arg106 (as donor) with 1*H*-pyrazol-2-ium moiety. In contrast, other chemical interactions were found on analyzing the binding modes of the lowest-scored poses for the four strongest-docking compounds (i.e., **11**, **29**, **42**, and **49**; Figure 5). These compounds resulted in the lowest mean docking scores (−10.48 to −11.26 kcal/mol range) and were found to be well-positioned into the COX-2 active site. However, they involve different orientations depending on the type of interactions. In this regard, residues Tyr341, Gln178, and Ser339 were found to be key polar contacts to stabilize the enzyme ligand complex for those compounds possessing a resorcinyl substituent, through a π-π-stacking for **29** and acting as an *H*-donor for **49**. Furthermore, the *p*-hydroxyphenyl group in **49** interacted with Trp373 via π-π-stacking. Psoralidin (**42**) exhibited another binding mode involving His75 as an *H*-donor to its phenolic OH at benzofuran moiety. Ala513 also interacted with phenolic OH at aryl moiety in 42. In addition, the methylenedioxy moiety in **11** was important to orient the molecule toward Arg499, acting as an *H*-donor acceptor. The combination of these structural features of such benzofurans could serve as an important starting point to design a novel series of COX-2 inhibitors.

### 3.3. Stability and Chemical Reactivity of the Best-Docked 2-Arylbenzofurans

**Figure 6:**
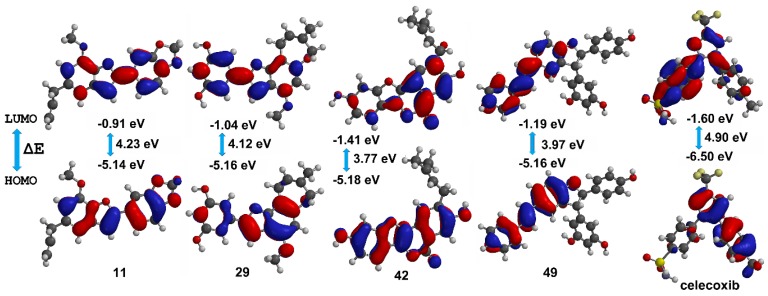
HOMO (highest occupied molecular orbital) and LUMO (lowest unoccupied molecular orbital) energies (in eV), HOMO-LUMO gaps (ΔE), and corresponding orbitals obtained by density functional theory (DFT) level for the best docked compounds (**11**, **29**, **42**, **49**, and celecoxib (cel)).

Frontier molecular orbitals HOMO and LUMO can reflect the successful biological interaction of ligands within the binding pocket of proteins [33]. In this sense, the DFT level-derived calculations of HOMO and LUMO energies were a suggestion of kinetic stability and chemical reactivity of these best docked compounds. The resulting B3LYP/6–311++G (2df, 2p) models are presented in Figure 6. These compounds exhibited lower HOMO-LUMO energy gaps to that of celecoxib and other 2-arylbenzofurans, which infer high stability but also high reactivity. Pearson’s correlation between COX-2 docking scores and HOMO-LUMO energy gaps for 2-arylbenzofurans **1**–**58** was also evaluated. A positive correlation among scores and energy gaps was found (0.812, *p* < 0.001), which indicated that the self-reactivity of compounds might influence the binding mode. Hence, among the best-docked compounds, **11** and **29** exhibited higher Vina scores and HOMO-LUMO energy gaps, whereas **42** and **49** showed an opposite behavior (Figure 6). This relationship can be rationalized since these energy gaps and eigenvalues defined the biological activity of ligands via high reactivity and good stability. Therefore, enzyme inhibition by these best-docked 2-arylbenzofurans might be afforded through an electron donating ability of test compounds with the enzyme-binding site pocket of COX-2.

### 3.4. Molecular Dynamics Studies of the Best-Docked 2-Arylbenzofurans

**Figure 7:**
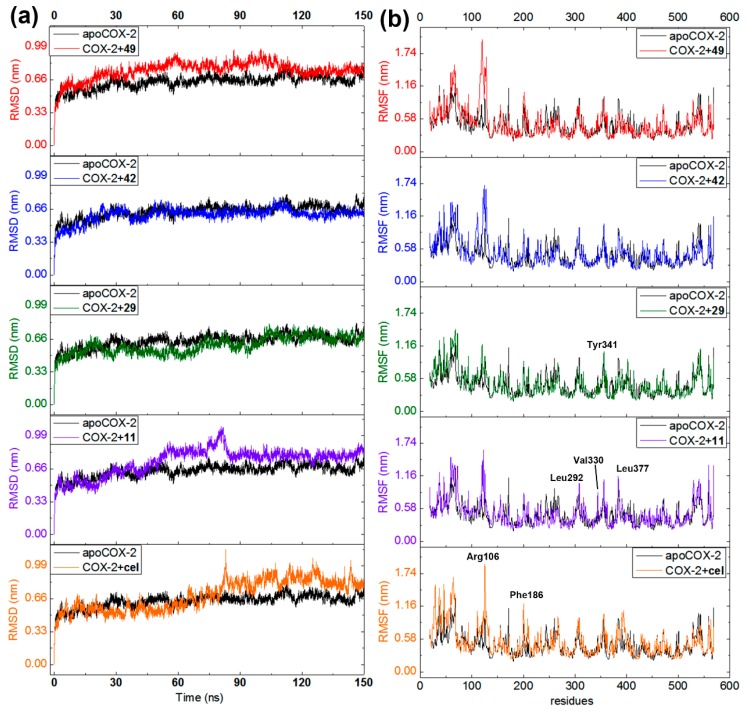
Molecular dynamics (MD) simulations during 150 ns for COX-2 apoenzyme (black line) and docked separately with **11** (purple line), **29** (green line), **42** (blue line), **49** (red line), and celecoxib (orange line). (**a**) Root mean square deviations (RMSD) along MD-simulated trajectories. (**b**) Root mean quadratic fluctuations (RMSF) of COX-2 residues (chain A) along MD-simulated trajectories.

In order to extend the information on the binding mode of the best docked compounds, 150-ns MD simulations were separately accomplished using the COX-2 (ES7) alone (apoenzyme) and docked distinctly with compounds **11**, **29**, **42**, **49**, and celecoxib. Ligand-enzyme trajectories for resulting complexes were examined through the variation of geometric properties over the time. Thus, RMSD of the COX-2 backbone reflected the receptor frame stability by computing the time-dependent distance (Å) among different positions of the atom set (Figure 7a). Apoenzyme exhibited a normal evolution during the simulation but revealed a slight perturbation at 30 and 50 ns (RMSD 0.50–0.65 nm). A normal stabilization was then reached throughout the rest of the MD simulation (RMSD 0.63 nm). The COX-2·celecoxib complex evolved typically but showed a late perturbation at 80 ns (until RMSD 0.99 nm). Although compounds **11** and **49** exhibited the most-perturbed profile, both complexes (with celecoxib and **11**) attained stability after 90 ns and **49** after 110 ns. The steady progress was maintained over the remaining time for these three ligands. On the other hand, compounds **29** and **42** exhibited the least-perturbed MD performance and the evolution was found to be similar to that of apoenzyme, which indicates good geometric properties on interacting with COX-2. The computed variations in RMSD values for these complexes showed that the COX-2 structure is affected differentially by the interaction with each ligand, which achieved a stable condition at the end of each MD simulation.

Fluctuations of the Cα atomic positions for each residue of the enzyme was explored through the root mean square fluctuation (RMSF). It was used to scrutinize the flexibility and secondary structure of the COX-2 enzyme under binding with the best-docked 2-arylbenzofurans. Thus, lower RMSF values implied constrained regions whereas higher RMSF values denoted more flexibility. All MD-simulated complexes showed similar performance (Figure 7b) involving fluctuations within the range of 0.2 and 1.6 nm. Such alterations were found to be near the formerly recognized crucial interactions (e.g., Arg106, Phe186, Leu292, Val330, Leu377, and Ser516). However, in comparison to the *apo*COX-2, the binding with test ligands did not substantially modify the flexibility of these regions to maintain an overall steady state (RMSF differences > 0.2 nm), excepting the most-fluctuating residues around Arg106. Therefore, the COX-2 inhibition mode of compounds **11**, **29**, **42**, and **49** might be interpreted through a complex stabilization, which is in agreement with the previously reported MD study for other known COX-2 inhibitors [34].

### 3.5. Binding-Free Energy Calculations of the Best-Docked 2-Arylbenzofurans

The binding-free energies (ΔG_bind_) for the best-docked compounds (i.e., **11**, **29**, **42**, **49**, and celecoxib) during the interaction of COX-2 for the last 40 ns of MD trajectory was estimated by the MM/PBSA approach to evaluate the global stability of the resulting ligand-enzyme complexes. The calculated binding energies are presented in Table 3. All five ligands exhibited negative binding energies. However, **42** showed comparable binding energy to that of celecoxib (−195.7 ± 4.5 kJ/mol vs. −197.8 ± 4.5 kJ/mol, respectively) and significant differences to that of other ligands, which justify the attained docking performance. The main contribution to the binding energy was due to van der Waal (ΔE_vdW_) energies (< −205 kJ/mol), which resulted in similar values among test compounds, while the contribution of electrostatic energy exhibited higher differences between them. Celecoxib and **49** exhibited the lowest electrostatic energy whereas 11 exhibited the highest one. In addition, the contribution of the polar solvation energy was found to be more unfavorable for **49** and celecoxib. The solvent accessible surface area (SASA) energy was similar for test ligands, even though compound **49** exhibited the highest ΔG_sasa_. Therefore, according to these results, electrostatic and polar solvation energy contributions could rationalize the difference in the binding mode of test ligands, which plays hydrophobic interactions as an important role in stabilization and even binding of test 2-arylbenzofurans within the active site of COX-2.

As a consequence, non-polar electrostatic interactions could be implied as the main driving force for the molecular recognition of COX-2 by test ligands. This fact was then confirmed after per-residue decomposition of the total binding energy of the simulated COX-2 ligand complexes. In this context, most binding energy contributing residues were found to be different depending on the ligand, as presented in Table 4. Regarding ligands, **42** exhibited similar binding energy to that of celecoxib (<−80 kJ/mol), which has the highest values among test ligands.

Regarding residues, Arg106, Leu338, and Val509 were found to be those residues that contribute the most (<−8 kJ/mol) to the binding energy for the COX-2·celecoxib complex (as described previously [34]), whereas Arg106 and Arg499 were observed for COX-2·11, Val335 for COX-2·29, Val335, Val509, and Ala513 for COX-2·42, and Glu510 and Ala513 for the COX-2·49 complex. Ser339 (<−2 kJ/mol) and Val509 (<−6 kJ/mol) were the common residues that contributed to the binding energy for all test ligands. Such residues with higher binding energy contributions represented those contact points for electrostatic interactions, as described in molecular docking and Cα atom fluctuations. Similar information was described for the binding mode of stilbene analogs within the active site of COX-2 [35].

### 3.6. In-Vitro COX-2 Activity of an in-House Collection of 2-Arylbenzofurans

The information on the potential as COX-2 inhibitors for the best-docked compounds (i.e., **11**, **29**, **42**, **49**) was expanded by measuring the IC_50_ (in μM) against COX-2 of an in-house library of 26 2-arylbenzofurans, which several of them were evaluated in a previous study [8]. The resulting IC_50_ values are presented in Table 5. Test compounds exhibited IC_50_ values in the range of 438 and 0.752 µM. In general, furocyclohexadienone and dihydrobenzofuran-containing compounds exhibited the highest IC_50_ values, except compound **49**, whose IC_50_ was 1.25 µM, which can be attributed to the resorcinyl and styryl substitutions at C3 and C5, respectively. These moieties influenced substantially to the binding of **49** since some interactions were favored with hydrophobic (i.e., styryl) and polar (i.e., resorcinyl) regions of the active site of COX-2. No significant differences were found for the IC_50_ values of compounds **11** and **29**, whose performances in molecular docking and molecular dynamics were very similar. The most active compound among the compound set was **42** (IC_50_ = 0.752 µM), which exhibited an interesting docking behavior (mean Vina score −11.25 ± 0.59 kcal/mol), the highest stability and reactivity (ΔE_HOMO-LUMO_ = 3.77 eV), and an excellent performance over the time for stabilizing the COX-2·42 complex. This predicted compound was previously reported to exhibit anti-inflammatory properties as a dual inhibitor of the expression of COX-2 and 5-LOX [32]. These results can be understood as an adequate validation of the molecular docking-derived discrimination to find COX-2 inhibitors based on 2-arylbenzofurans. Previously mentioned most-active compounds also exhibited favorable ADMET properties (calculated using the FAF-Drugs4 web server [36]), which is afforded as accepted since the overall result with no Lipinski’s rule violation, good solubility, and oral bioavailability (Table A3).

#### Comparative Molecular Field Analysis (CoMFA)

A CoMFA was applied to correlate molecular interaction fields (MIF) and COX-2 inhibition in order to consistently rationalize the inhibitory activity of 2-arylbenzofurans regarding electrostatic and steric properties [38] as well as describing and predicting some structural requirements for COX-2 inhibition by 2-arylbenzofurans. For this purpose, the best-docked pose of compound **11** was used as a template to outline tethers and overlay the test compounds (Table 5), due to the structural features. Aligned structures and experimental data of COX-2 inhibition (as *p*IC_50_ values) were segmented into a training set (70%) to create the respective CoMFA model and a test set (30%) for the external validation [38]. MIFs were computed by means of electrostatic and steric probes. Partial least squares (PLS) regression was performed (employing up to five PLS components). Linear relationships between MIF fluctuations according to variations of the experimental *p*IC_50_ were accomplished. Thus, the best model achieved good correlation between MIF values and experimental *p*IC_50_ of test compounds using four PLS components, which comprises a correlation coefficient r^2^ = 0.816, a F-test = 162.321, cross-validated LOO coefficient q^2^_LOO_ = 0.766, and a cross-validated LMO coefficient q^2^_LMO_ = 0.701. These parameters were found to be enough for a statistically robust, predictive model [39]. The adequate correlation was examined through a *Y*-scrambling procedure [40]. Hence, 30 scramblings and 10 runs were employed. After that, no correlation appeared since the models dropped significantly (R_scr_^2^ and Q_scr_^2^ < 0.3). This fact suggested the model was not reached as a consequence of a coincidental correlation. Therefore, this CoMFA-based model predicted the activity for the entire compound set, expressed as *p*IC_50_ (pred) (Table 5), which demonstrated a reasonable correlation for both training and test datasets.

**Figure 8:**
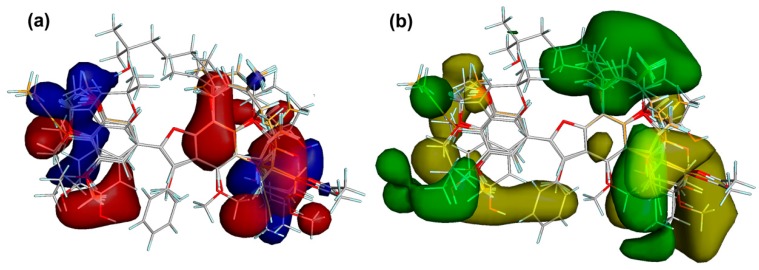
Contour maps from the field contributions from the comparative molecular field analysis (CoMFA). (**a**) Electrostatic contour maps. Blue and red contours depict zones where positive and negative charges, respectively, will favor the COX-2 inhibitory activity. (**b**) Steric contour maps. Yellow and green contours indicate regions where a bulky group is unfavorable and favorable, respectively, for COX-2 inhibition.

Steric and electrostatic field outputs (stdev * coeff) reached values of 31.1% and 37.4%, respectively. Figure 8 showed the corresponding translated contour surfaces from the field contributions. The electrostatic field contour map (Figure 8a) displayed the positively and negatively-charged regions to favor COX-2 inhibition as blue and red contours, respectively. Accordingly, phenolic hydroxyl groups at C4′ and C7, resorcinyl moiety, and electron-withdrawing groups at C6 enhanced the COX-2 activity to interact with some residues located in the polar region of the active site of this enzyme (e.g., Hiz75, Arg499, and Gln178), as observed for compounds **29**, **42**, and **49**. Alkyl (such as allyl, styryl, and prenyl) and methoxyl substitutions on 2-aryl moiety also favored the COX-2 inhibition (e.g., compounds **11** and **42**) to interact positively or negatively with residues in the hydrophobic zone of the COX-2 active site (i.e., Val102, Leu103, and Leu345).

On the other side, the enzymatic inhibitory activity might also be enhanced/decreased by the presence of bulky groups at benzofuran and 2-aryl moieties, as observed in the steric field contour maps (Figure 8b). Unfavorable and favorable zones by steric effects were then depicted as yellow and green contours, respectively. COX-2 inhibitory activity could be improved if a bulky steric group is located on C3′, C5, and C7, such as substituted prenyl groups and dihydropyran ring, as observed in most active compounds **29** and **42**. In contrast, a bulky group in C2′ (e.g., hydroxypropenyl) and C3 (e.g., phenyl group) negatively influenced the enzymatic inhibition. Consequently, the CoMFA model indicated that the particular presence of both bulky and electronegative substituents (oriented sterically and electrostatically toward polar and hydrophobic zones within the active site) could be considered as important structural features to improve the COX-2 inhibitory activity by 2-arylbenzofurans.

## 4. Conclusions

An in-silico study was primarily performed on a custom-made library comprising 58 benzofuran-containing compounds of natural origin in order to discriminate binders and non-binders of COX-2. Thus, those 2-arylbenzofurans, having an additional ring (such as oxepine) or bulky substitutions in benzofuran or aryl moieties, can be considered as potential hits for COX-2 inhibition due to the favorable characteristics for an adequate binding within the active site of this enzyme. In this regard, the best-docked compounds exhibited the best performance in MD simulations, the most-favorable binding energies, and the lowest in-vitro IC_50_ values for COX-2 inhibition. Consequently, the molecular docking behavior of 2-arylbenzofurans within the active site of this enzyme might be used as an appropriate strategy to predict/analyze their binding mode and potentiality as COX-2 inhibitors. Lastly, the findings through the CoMFA model demonstrated a structure-activity relationship, which shows some crucial structural requirements for enhanced enzyme inhibition. These structural features must be oriented sterically and electrostatically toward both polar and hydrophobic zones within the active site to promote crucial interactions. These oriented interactions are the main driving force for the molecular recognition of COX-2 by test ligands. In this context, four hits (i.e., **11**, **29**, **42**, and **49**) are then proposed as lead structures for the development of COX-2 inhibitors. Therefore, such information should be conserved during further studies by focusing on the development of COX-2 inhibitors based on 2-arylbenzofurans.

## Figures and Tables

**Table 1:** Data of the crystal structures of COX-2.

Code *^a^*	PDB ID *^b^*	Resolution [Å]	Bound	Source	Reference *^c^*
ES1	1CVU	2.40	arachidonic acid	*Mus musculus*	[10]
ES2	5COX	3.00	unbounded	*M. musculus*	[11]
ES3	4COX	2.90	indomethacin	*M. musculus*	[11]
ES4	1CX2	3.00	SC-558	*M. musculus*	[11]
ES5	1PXX	2.90	diclofenac	*M. musculus*	[12]
ES6	3NT1	1.73	naproxen	*M. musculus*	[13]
ES7	3LN1	2.40	celecoxib	*M. musculus*	[14]
ES8	4RS0	2.81	*S*-ibuprofen	*M. musculus*	[15]
ES9	4PH9	1.81	*rac*-ibuprofen	*M. musculus*	[16]

*^a^*ES = Enzyme Structure. *^b^* Protein Data Bank (PDB) code. *^c^* Reference including the active site.

**Table 2:** Global mean values of Vina scores, relative standard deviation percentage (%RSD), and root mean square deviation (RMSD) (in Å) of 2-arylbenzofurans docked with cyclooxygenase-2 (COX-2) structures.

No.	GMVS *^a^*	%RSD *^b^*	RMSD *^c^*	No.	GMVS *^a^*	%RSD *^b^*	RMSD *^c^*	No.	GMVS *^a^*	%RSD *^b^*	RMSD *^c^*
**1**	−8.63 ± 0.50	5.85	0.521	**23**	−9.37 ± 0.83	8.88	0.513	**45**	−9.21 ± 0.93	10.06	0.335
**2**	−9.19 ± 0.84	9.14	0.307	**24**	−9.23 ± 0.89	9.67	0.626	**46**	−8.79 ± 1.11	12.60	0.475
**3**	−8.10 ± 0.94	11.61	0.319	**25**	−8.50 ± 1.35	15.85	0.493	**47**	−8.67 ± 1.14	13.14	0.345
**4**	−8.43 ± 0.50	5.90	0.712	**26**	−8.78 ± 1.18	4.88	0.574	**48**	−8.79 ± 1.15	13.11	0.333
**5**	−8.50 ± 0.53	6.25	0.404	**27**	−10.16 ± 0.50	13.40	0.611	**49**	−11.26 ± 0.81	5.48	0.496
**6**	−8.14 ± 0.74	9.09	0.549	**28**	−10.47 ± 0.46	9.38	0.711	**50**	−8.48 ± 1.58	18.66	0.520
**7**	−8.43 ± 0.52	6.19	0.531	**29**	−10.49 ± 0.43	4.14	0.450	**51**	−10.37 ± 0.57	7.16	0.621
**8**	−8.49 ± 0.39	4.58	0.576	**30**	−10.42 ± 0.54	12.75	0.790	**52**	−8.18 ± 0.92	11.30	0.452
**9**	−8.92 ± 0.38	4.30	0.605	**31**	−9.49 ± 1.21	5.21	0.471	**53**	−9.07 ± 0.72	5.60	0.467
**10**	−8.51 ± 0.46	9.03	0.490	**32**	−8.84 ± 1.66	18.80	0.564	**54**	−10.39 ± 0.58	7.95	0.842
**11**	−10.76 ± 0.27	5.40	0.458	**33**	−8.32 ± 1.42	17.12	0.316	**55**	−8.72 ± 0.49	5.61	0.433
**12**	−8.99 ± 0.98	10.88	0.494	**34**	−8.63 ± 1.74	20.14	0.526	**56**	−8.49 ± 0.45	5.32	0.368
**13**	−9.24 ± 1.20	12.96	0.578	**35**	−8.70 ± 1.23	14.15	0.548	**57**	−7.77 ± 1.38	17.81	0.307
**14**	−8.58 ± 0.57	6.59	0.529	**36**	−9.02 ± 0.79	8.71	0.646	**58**	−8.46 ± 0.67	7.98	0.367
**15**	−8.93 ± 1.17	13.08	0.602	**37**	−9.03 ± 0.58	6.41	0.534	**AA**	−7.98 ± 0.37	4.60	1.329
**16**	−9.09 ± 1.05	11.53	0.367	**38**	−8.73 ± 0.58	6.65	0.426	**CEL**	−11.23 ± 0.62	5.52	0.488
**17**	−8.87 ± 0.86	9.75	0.516	**39**	−10.2 ± 0.69	6.79	0.443	**DCL**	−8.33 ± 0.30	3.65	0.318
**18**	−8.77 ± 1.24	14.14	0.425	**40**	−10.3 ± 0.59	7.42	0.547	**IMD**	−9.67 ± 0.79	8.13	0.449
**19**	−9.02 ± 0.71	7.89	0.493	**41**	−9.70 ± 0.72	5.74	0.447	**NPX**	−8.66 ± 0.29	3.32	0.376
**20**	−9.11 ± 1.20	13.18	0.596	**42**	−11.25 ± 0.59	5.28	0.335	**SC558**	−11.09 ± 0.57	5.13	0.412
**21**	−9.33 ± 0.89	9.55	0.574	**43**	−9.32 ± 0.75	8.01	0.322	***S*−IBP**	−7.71 ± 0.33	4.22	0.638
**22**	−9.04 ± 0.84	9.25	0.534	**44**	−9.13 ± 0.98	10.73	0.320	***R*−IBP**	−7.71 ± 0.27	3.52	0.784

*^a^* Global mean values of Vina scores (GMVS) (kcal/mol) ± standard deviation (SD) for each test 2-arylbenzofuran docked with nine different COX-2 structures. *^b^* Relative Standard Deviation (RSD) percentages for each test 2-arylbenzofuran. *^c^* Mean RMSD values for each test 2-arylbenzofuran.

**Table 3:** Binding-free and related energies calculated using molecular mechanic/Poisson Boltzmann surface area (MM/PBSA) method for test compounds.

Ligands	ΔE_vdW_ *^a^*	ΔE_ele_ *^b^*	ΔG_sol_ *^c^*	ΔG_sasa_ *^d^*	ΔG_bind_ *^e^*
**11**	−211.7 ± 2.5	−96.3 ± 1.6	185.6 ± 7.5	−19.0 ± 0.2	−141.5 ± 5.8
**29**	−221.2 ± 5.4	−109.2 ± 3.9	175.6 ± 2.7	−20.2 ± 0.3	−175.0 ± 4.3
**42**	−205.6 ± 3.5	−102.5 ± 3.6	132.2 ± 4.8	−19.8 ± 0.2	−195.7 ± 4.5
**49**	−226.9 ± 4.5	−120.8 ± 4.6	189.2 ± 3.7	−24.4 ± 0.4	−182.9 ± 2.0
**celecoxib**	−225.5 ± 8.9	−138.2 ± 5.7	184.5 ± 8.5	−18.6 ± 0.3	−197.8 ± 7.5

*^a^* Van der Waal (vdW) energy. *^b^* Electrostatic energy. *^c^* Polar solvation energy. *^d^* Solvent accessible surface area (SASA) energy. *^e^* Binding-free energy. Energies are expressed as mean values (in kJ/mol) ± standard deviation.

**Table 4:** Molecular mechanic/Poisson Boltzmann surface area (MM/PBSA) per-residue decomposition energies for selected residues of those complexes between COX-2 and **11**, **29**, **42**, **49**, and celecoxib using the last 40-ns molecular dynamics (MD) simulations.

Residue	11	29	42	49	Cel
Val74	−0.22 ± 0.42	−0.15 ± 0.07	−0.08 ± 0.05	−4.12 ± 0.76	−0.37 ± 0.08
His75	−0.57 ± 0.40	−2.12 ± 0.58	−0.24 ± 0.82	−2.26 ± 0.73	−1.93 ± 0.37
Leu103	−1.07 ± 0.15	−1.02 ± 0.26	−3.63 ± 0.83	−0.77 ± 0.05	−0.54 ± 0.04
Arg106	−9.64 ± 3.76	−0.35 ± 0.51	−6.67 ± 0.99	−0.36 ± 2.84	−8.94 ± 1.33
Gln178	−1.77 ± 0.30	−1.68 ± 0.32	−1.48 ± 0.21	−0.53 ± 0.29	−4.97 ± 0.38
Val330	−2.08 ± 0.06	−0.08 ± 0.08	−2.13 ± 0.38	−0.02 ± 0.02	−0.28 ± 0.15
Val335	−5.43 ± 0.46	−9.27 ± 0.34	−11.49 ± 0.72	−2.17 ± 0.61	−4.65 ± 0.81
Leu338	−1.41 ± 0.90	−3.29 ± 1.09	−4.13 ± 0.74	−5.39 ± 0.77	−8.87 ± 1.19
Ser339	−2.59 ± 0.81	−4.39 ± 0.56	−4.86 ± 0.39	−4.98 ± 0.91	−6.37 ± 1.01
Tyr341	−0.07 ± 0.63	−4.58 ± 0.55	−1.56 ± 0.54	−1.18 ± 0.75	−3.01 ± 0.52
Leu345	−2.29 ± 0.41	−1.59 ± 0.26	−1.42 ± 0.43	−0.85 ± 0.15	−1.80 ± 0.08
Leu370	−0.16 ± 0.04	−0.13 ± 0.03	−0.17 ± 0.03	−1.91 ± 0.15	−1.32 ± 0.38
Tyr371	−0.08 ± 0.11	−0.55 ± 0.16	−0.58 ± 0.32	−0.46 ± 0.26	−1.49 ± 0.92
Arg499	−5.32 ± 1.33	−3.83 ± 1.39	−1.35 ± 1.41	−6.24 ± 1.26	−7.90 ± 1.17
Phe504	−3.12 ± 0.44	−2.93 ± 0.91	−2.37 ± 0.51	−2.41 ± 0.48	−6.23 ± 1.03
Val509	−6.17 ± 0.58	−6.41 ± 1.03	−8.11 ± 0.33	−7.36 ± 0.91	−9.91 ± 0.43
Glu510	−3.01 ± 1.03	−0.61 ± 0.99	−1.27 ± 0.55	−13.3 ± 1.76	−6.89 ± 0.58
Gly512	−0.61 ± 0.17	−1.32 ± 0.25	−1.07 ± 0.07	−3.68 ± 0.56	−3.29 ± 0.66
Ala513	−3.84 ± 1.03	−5.55 ± 0.71	−9.86 ± 0.24	−7.57 ± 0.37	−5.09 ± 0.83
Ser516	−2.17 ± 0.91	−0.52 ± 0.61	−2.48 ± 0.58	−2.17 ± 0.71	−2.78 ± 0.49
Leu517	−1.50 ± 1.24	−1.36 ± 0.58	−5.96 ± 0.59	−1.54 ± 0.28	−1.89 ± 0.32
Ligand	−60.9 ± 1.5	−61.5 ± 1.97	−83.4 ± 2.44	−69.6 ± 2.24	−92.6 ± 3.70

**Table 5:** In-vitro COX-2 inhibition of an in-house collection (*n* = 26) of 2-arylbenzofurans.

No	COX-2 IC_50_	pIC_50_ *^a^*	pIC_50_ *^b^*	No	COX-2 IC_50_	pIC_50_ *^a^*	pIC_50_ *^b^*	No	COX-2 IC_50_	pIC_50_ *^a^*	pIC_50_ *^b^*
	(µM)	(exp)	(pred)		(µM)	(exp)	(pred)		(µM)	(exp)	(pred)
**2**	16.7 *^c^*	3.78	4.04	**29**	3.75	4.43	3.94	**49**	1.25	4.90	4.71
**3**	13.2 *^c^*	3.49	3.35	**38**	158	2.80	2.86	**50**	45.6	3.34	3.21
**8**	28.7	3.54	3.72	**42**	0.752	5.12	5.18	**51**	10.5	3.98	4.14
**9**	119	2.92	3.32	**43 *^c^***	102	2.99	3.45	**54**	10.9	3.96	3.84
**11**	3.27 *^c^*	4.49	4.09	**44 *^c^***	26.2	3.58	3.12	**55**	12.6	3.90	4.17
**12**	4.33 *^c^*	4.36	3.98	**45 *^c^***	52.7	3.28	2.95	**56 *^c^***	125	2.90	2.51
**13**	8.56 *^c^*	4.07	4.46	**46 *^c^***	32.9	3.48	3.59	**57 *^c^***	438	2.36	2.21
**14**	7.37 *^c^*	4.13	4.55	**47 *^c^***	71.5	3.15	3.55	**58 *^c^***	240	2.62	2.72
**17**	12.3 *^c^*	3.91	4.22	**48 *^c^***	29.3	3.53	3.78	**cel**	0.102	-	-

*^a^p*IC_50_(exp) = −log(IC_50_(exp) in M), *^b^* predicted from CoMFA model. *^c^* Compounds previously evaluated for COX-2 inhibitory activity [8,37].

## Appendix A

**Table A1:** Common names of test 2-arylbenzofuran-related compounds.

No.	Common Name	Reference	No.	Common Name	Reference
**1**	moracin Y	[7]	**30**	moracin L	[7]
**2**	ocophyllal A	[8]	**31**	moracin U	[7]
**3**	ocophyllal B	[8]	**32**	moracin P	[7]
**4**	moracin A	[7]	**33**	moracin Q	[7]
**5**	moracin B	[7]	**34**	moracin V	[7]
**6**	moracin F	[7]	**35**	moracin O	[7]
**7**	moracin J	[7]	**36**	moracin X	[7]
**8**	moracin M	[7]	**37**	substituted benzofuran 2 *^b^*	[41]
**9**	(+)-obtusafuran	[42]	**38**	isoparvifuran	[43]
**10**	substituted benzofuran 1 *^a^*	[44]	**39**	moracin D	[7]
**11**	9′-*nor*-7,8-dehydro-isolicarin-B	[8]	**40**	moracin K	[7]
**12**	egonol	[37]	**41**	kanzonol U	[45]
**13**	acetylegonol	[37]	**42**	psoralidin	[46]
**14**	homoegonol	[37]	**43**	liliflol B	[8]
**15**	zanthocapensol	[47]	**44**	(−)-licarin B	[8]
**16**	zanthocapensate	[47]	**45**	(+)-licarin B	[8]
**17**	acetylhomoegonol	[37]	**46**	(−)-licarin A	[8]
**18**	moracin C	[7]	**47**	(+)-licarin A	[8]
**19**	moracin I	[7]	**48**	(+)-acuminatin	[8]
**20**	moracin N	[7]	**49**	maximol	[48]
**21**	moracin S	[7]	**50**	picrasmalignan A	[49]
**22**	moracin T	[7]	**51**	salvianolic acid B	[50]
**23**	dinklagein A	[9]	**52**	substituted pterocarpan 1 *^c^*	[51]
**24**	dinklagein B	[9]	**53**	substituted pterocarpan 2 *^d^*	[51]
**25**	moracin R	[7]	**54**	(+)-glyceollin I	[52]
**26**	moracin Z	[7]	**55**	sulfuretin	[53]
**27**	moracin W	[7]	**56**	(+)-mirandin A	[8]
**28**	moracin G	[7]	**57**	kadsurenone	[8]
**29**	moracin H	[7]	**58**	(+)-denudatin B	[8]

*^a^* 6-hydroxy-2-(2-hydroxy-4-methoxyphenyl)-benzofuran, *^b^* ethyl 2-methyl-5-phenoxybenzofuran-3-carboxylate, *^c^* 2*S*,3*S*-3,8-dihydroxy-2,9-dimethoxy-pterocarpan, *^d^* 2*R*,3*R*-3,8-dihydroxy-2,9-dimethoxy-pterocarpan.

**Table A2:** Molecular docking results (Vina scores) of 2-arylbenzofuran-related compounds with COX-2.

No.	ES1 (1CVU) *^a^*		ES2 (1CX2) *^a^*		ES3 (4COX) *^a^*		ES4 (5COX) *^a^*		ES5 (1PXX) *^a^*		ES6 (3NT1) *^a^*		ES7 (3LN1) *^a^*		ES8 (4RS0) *^a^*		ES9 (4PH9) *^a^*	
	VS ± SD *^b^*	RMS *^c^*	VS ± SD *^b^*	RMS *^c^*	VS ± SD *^b^*	RMS *^c^*	VS ± SD *^b^*	RMS *^c^*	VS ± SD *^b^*	RMS *^c^*	VS± SD *^b^*	RMS *^c^*	VS ± SD *^b^*	RMS *^c^*	VS ± SD *^b^*	RMS *^c^*	VS ± SD *^b^*	RMS *^c^*
**1**	−8.8 ± 0.1	0.435	−9.1 ± 0.1	0.337	−8.6 ± 0.1	0.808	−8.4 ± 0.2	0.566	−8.5 ± 0.2	0.361	−8.3 ± 0.1	0.879	−9.7 ± 0.2	0.699	−8.1 ± 0.1	0.299	−8.2 ± 0.1	0.303
**2**	−9.6 ± 0.2	0.308	−8.9 ± 0.2	0.225	−10.0 ± 0.2	0.319	−9.7 ± 0.1	0.269	−9.7 ± 0.1	0.240	−8.3 ± 0.2	0.282	−10.2 ± 0.1	0.674	−8.5 ± 0.1	0.183	−7.8 ± 0.1	0.264
**3**	−7.3 ± 0.2	0.239	−9.0 ± 0.2	0.272	−8.9 ± 0.2	0.255	−8.6 ± 0.1	0.848	−8.7 ± 0.1	0.441	−7.6 ± 0.1	0.264	−8.9 ± 0.1	0.178	−7.6 ± 0.1	0.165	−6.3 ± 0.2	0.210
**4**	−8.7 ± 0.1	0.933	−8.7 ± 0.1	0.877	−8.5 ± 0.1	0.936	−8.1 ± 0.4	0.492	−8.3 ± 0.2	0.960	−8.2 ± 0.2	0.358	−9.5 ± 0.1	0.447	−8.1 ± 0.2	0.334	−7.8 ± 0.1	1.075
**5**	−8.6 ± 0.2	0.973	−9.1 ± 0.1	0.262	−8.1 ± 0.1	0.699	−8.4 ± 0.3	0.311	−8.4 ± 0.1	0.355	−8.2 ± 0.2	0.238	−9.6 ± 0.1	0.128	−8.1 ± 0.1	0.325	−8.0 ± 0.1	0.344
**6**	−8.5 ± 0.1	0.342	−8.3 ± 0.1	0.344	−8.2 ± 0.2	0.587	−8.1 ± 0.1	0.288	−8.0 ± 0.1	0.885	−7.3 ± 0.1	0.927	−9.8 ± 0.2	0.038	−7.4 ± 0.1	0.351	−7.7 ± 0.1	1.182
**7**	−8.5 ± 0.1	0.346	−9 ± 0.2	0.352	−7.9 ± 0.2	0.279	−8.4 ± 0.1	0.880	−8.2 ± 0.1	0.334	−8.4 ± 0.1	0.374	−9.5 ± 0.1	0.673	−8.0 ± 0.1	0.344	−8.0 ± 0.1	1.201
**8**	−8.5 ± 0.1	0.432	−8.8 ± 0.1	0.854	−8.4 ± 0.1	0.924	−8.5 ± 0.1	0.869	−8.4 ± 0.2	0.385	−8.4 ± 0.1	0.400	−9.3 ± 0.2	0.370	−8.2 ± 0.1	0.650	−7.9 ± 0.1	0.298
**9**	−8.7 ± 0.2	0.869	−8.8 ± 0.2	0.503	−8.9 ± 0.2	0.986	−9.8 ± 0.2	0.304	−9.0 ± 0.1	0.308	−8.6 ± 0.1	0.455	−9.2 ± 0.2	0.734	−8.6 ± 0.1	0.664	−8.7 ± 0.1	0.619
**10**	−8.4 ± 0.1	0.346	−8.8 ± 0.1	0.314	−8.7 ± 0.2	0.358	−8.6 ± 0.1	0.707	−8.8 ± 0.1	0.329	−8.1 ± 0.1	0.490	−9.3 ± 0.2	0.941	−8.1 ± 0.1	0.362	−7.8 ± 0.1	0.274
**11**	−10.6 ± 0.2	0.276	−10.5 ± 0.4	0.802	−10.8 ± 0.2	0.299	−10.9 ± 0.1	0.924	−11.3 ± 0.1	0.178	−10.8 ± 0.2	0.661	−10.6 ± 0.1	0.935	−10.4 ± 0.2	0.118	−10.9 ± 0.2	0.220
**12**	−9.2 ± 0.1	0.395	−9.9 ± 0.4	0.797	−9.7 ± 0.1	0.499	−9.2 ± 0.1	0.789	−9.2 ± 0.2	0.324	−6.9 ± 0.1	0.252	−9.9 ± 0.2	0.860	−8.0 ± 0.1	0.213	−8.9 ± 0.1	0.313
**13**	−9.8 ± 0.1	0.652	−10.6 ± 0.2	0.662	−10.6 ± 0.1	0.287	−9.5 ± 0.1	0.422	−9.6 ± 0.1	0.273	−7.6 ± 0.3	0.255	−9.8 ± 0.2	0.594	−7.4 ± 0.3	1.110	−8.3 ± 0.2	0.947
**14**	−8.7 ± 0.3	1.054	−8.7 ± 0.2	0.577	−9.4 ± 0.2	0.362	−8.2 ± 0.1	0.716	−8.8 ± 0.1	0.285	−7.7 ± 0.1	0.307	−9.0 ± 0.2	0.918	−7.8 ± 0.1	0.275	−8.9 ± 0.1	0.267
**15**	−9.6 ± 0.1	1.134	−10.3 ± 0.2	0.605	−9.0 ± 0.1	0.327	−9.3 ± 0.2	0.318	−9.4 ± 0.1	0.295	−6.9 ± 0.3	0.277	−9.8 ± 0.1	0.829	−7.1 ± 0.1	1.393	−9.0 ± 0.2	0.244
**16**	−9.6 ± 0.1	0.276	−10.6 ± 0.2	0.767	−9.6 ± 0.1	0.433	−9.1 ± 0.3	0.258	−9.6 ± 0.1	0.477	−7.4 ± 0.2	0.252	−9.6 ± 0.2	0.477	−7.5 ± 0.2	0.107	−8.8 ± 0.1	0.260
**17**	−9.2 ± 0.1	0.587	−9.2 ± 0.3	0.572	−10.2 ± 0.2	0.424	−8.9 ± 0.2	0.414	−9.4 ± 0.1	0.253	−7.6 ± 0.1	0.293	−9.4 ± 0.1	0.573	−7.7 ± 0.1	0.555	−8.2 ± 0.1	0.974
**18**	−9.0 ± 0.1	0.485	−8.7 ± 0.3	0.350	−9.5 ± 0.1	0.316	−9.4 ± 0.1	0.373	−9.2 ± 0.1	0.314	−7.7 ± 0.1	0.351	−11.0 ± 0.1	0.827	−7.0 ± 0.5	0.391	−7.4 ± 0.2	0.418
**19**	−9.3 ± 0.2	0.385	−8.5 ± 0.1	0.353	−9.2 ± 0.2	0.768	−8.9 ± 0.1	0.872	−8.6 ± 0.1	0.386	−8.5 ± 0.2	0.860	−10.7 ± 0.1	0.144	−9.1 ± 0.1	0.311	−8.4 ± 0.1	0.360
**20**	−9.5 ± 0.1	1.199	−9.6 ± 0.2	0.900	−9.6 ± 0.2	0.317	−9.1 ± 0.2	0.333	−9.8 ± 0.2	0.325	−7.4 ± 0.1	0.924	−11.1 ± 0.1	0.586	−7.3 ± 0.1	0.393	−8.6 ± 0.1	0.388
**21**	−9.9 ± 0.1	0.361	−9.6 ± 0.4	0.913	−9.2 ± 0.1	0.673	−10.3 ± 0.1	0.789	−9.0 ± 0.1	0.344	−8.1 ± 0.1	0.845	−10.8 ± 0.2	0.532	−8.6 ± 0.1	0.305	−8.5 ± 0.1	0.403
**22**	−9.1 ± 0.1	0.274	−10.0 ± 0.2	0.489	−8.8 ± 0.1	0.520	−8.9 ± 0.1	0.394	−9.1 ± 0.1	0.343	−8.4 ± 0.1	0.331	−10.7 ± 0.3	0.958	−8.1 ± 0.1	1.139	−8.3 ± 0.1	0.359
**23**	−9.4 ± 0.2	0.345	−9.6 ± 0.3	0.563	−9 ± 0.1	0.569	−10.4 ± 0.2	0.338	−9.2 ± 0.1	0.784	−8.5 ± 0.2	0.632	−10.9 ± 0.1	0.358	−8.9 ± 0.2	0.478	−8.4 ± 0.2	0.412
**24**	−9.3 ± 0.2	0.756	−9.2 ± 0.3	0.478	−9.2 ± 0.2	0.656	−10.6 ± 0.2	0.589	−9.4 ± 0.1	0.779	−8.2 ± 0.1	0.456	−10.5 ± 0.3	0.665	−8.7 ± 0.2	0.851	−8 ± 0.2	0.458
**25**	−8.4 ± 0.1	0.372	−8.7 ± 0.3	0.391	−9.3 ± 0.1	0.401	−8.7 ± 0.2	0.885	−9.6 ± 0.1	0.953	−6.7 ± 0.3	0.382	−10.8 ± 0.1	0.249	−7.6 ± 0.2	0.391	−6.7 ± 0.1	0.412
**26**	−9.0 ± 0.1	0.950	−9.5 ± 0.2	0.919	−9.4 ± 0.1	0.473	−8.8 ± 0.3	0.776	−9.3 ± 0.1	0.322	−6.8 ± 0.7	0.395	−10.7 ± 0.2	0.719	−7.5 ± 0.1	0.554	−8.0 ± 0.1	0.392
**27**	−10.7 ± 0.1	0.535	−9.7 ± 0.3	0.561	−9.9 ± 0.1	0.977	−10.0 ± 0.3	0.571	−9.7 ± 0.5	0.823	−10.0 ± 0.1	0.507	−11.1 ± 0.3	0.413	−9.8 ± 0.2	0.373	−10.5 ± 0.2	0.403
**28**	−10.7 ± 0.1	0.859	−9.8 ± 0.1	0.344	−10.4 ± 0.1	0.275	−10.3 ± 0.1	0.336	−9.9 ± 0.1	0.329	−10.4 ± 0.5	0.303	−11.3 ± 0.1	0.660	−10.6 ± 0.1	0.290	−10.8 ± 0.1	0.303
**29**	−10.3 ± 0.2	0.351	−10.9 ± 0.5	0.692	−10.3 ± 0.1	0.328	−10.0 ± 0.1	0.350	−10.1 ± 0.1	0.336	−10.5 ± 0.2	0.288	−11.4 ± 0.1	0.883	−10.6 ± 0.1	0.257	−10.3 ± 0.5	3.621
**30**	−10.2 ± 0.1	0.864	−10.5 ± 0.2	0.329	−10.2 ± 0.1	0.472	−10.4 ± 0.1	0.366	−9.3 ± 0.2	0.363	−10.4 ± 0.4	0.346	−11.3 ± 0.1	0.604	−10.7 ± 0.1	0.354	−10.8 ± 0.1	0.355
**31**	−9.1 ± 0.1	0.340	−9.9 ± 0.2	0.372	−10.0 ± 0.4	0.396	−9.7 ± 0.1	0.404	−10.7 ± 0.1	0.932	−7.2 ± 0.3	0.353	−11.3 ± 0.1	0.622	−8.7 ± 0.1	0.404	−8.8 ± 0.1	0.419
**32**	−8.8 ± 0.1	0.915	−9.3 ± 0.1	0.316	−10.3 ± 0.1	0.353	−9.7 ± 0.1	0.918	−9.5 ± 0.1	0.335	−6.6 ± 1.8	0.547	−11.3 ± 0.2	0.999	−6.3 ± 0.2	0.300	−7.8 ± 0.1	0.390
**33**	−7.0 ± 0.1	0.311	−9.2 ± 0.2	0.301	−10.2 ± 0.2	0.302	−9.0 ± 0.1	0.297	−9.4 ± 0.1	0.263	−7.1 ± 0.1	0.213	−9.6 ± 0.2	0.662	−6.4 ± 0.1	0.206	−7.0 ± 0.1	0.288
**34**	−8.6 ± 0.2	0.340	−9.1 ± 0.1	0.359	−10.3 ± 0.1	0.409	−9.8 ± 0.3	0.931	−9.0 ± 0.1	0.672	−6.4 ± 1.7	0.380	−11.2 ± 0.1	0.918	−6.3 ± 0.1	0.364	−7.0 ± 0.1	0.358
**35**	−8.2 ± 0.2	0.481	−9.6 ± 0.1	0.314	−10.0 ± 0.1	0.353	−9.2 ± 0.1	0.358	−9.8 ± 0.1	0.893	−7.2 ± 0.1	0.381	−9.8 ± 0.1	0.683	−6.9 ± 0.2	0.352	−7.6 ± 0.1	1.113
**36**	−9.1 ± 0.1	0.792	−9.7 ± 0.3	0.321	−9.0 ± 0.1	0.507	−8.3 ± 0.3	0.898	−9.1 ± 0.1	0.735	−8.2 ± 0.1	0.296	−10.7 ± 0.2	0.815	−8.6 ± 0.1	0.302	−8.5 ± 0.1	1.144
**37**	−9.4 ± 0.1	0.592	−9.4 ± 0.1	0.474	−9.3 ± 0.1	0.630	−9.1 ± 0.2	0.887	−9.5 ± 0.1	0.773	−7.9 ± 0.1	0.225	−9.6 ± 0.2	0.865	−8.4 ± 0.1	0.140	−8.7 ± 0.2	0.216
**38**	−9.0 ± 0.1	0.463	−8.6 ± 0.2	0.839	−8.8 ± 0.2	0.502	−9.4 ± 0.1	0.301	−8.5 ± 0.1	0.486	−8.2 ± 0.1	0.327	−9.8 ± 0.1	0.049	−8.2 ± 0.1	0.538	−8.1 ± 0.1	0.328
**39**	−10.1 ± 0.1	0.306	−10.3 ± 0.5	0.334	−9.7 ± 0.1	0.922	−9.2 ± 0.1	0.299	−10.2 ± 0.1	0.312	−10.2 ± 0.3	0.339	−11.8 ± 0.2	0.826	−10.2 ± 0.1	0.316	−10.1 ± 0.2	0.331
**40**	−10.0 ± 0.1	0.562	−10.5 ± 0.1	0.283	−10.1 ± 0.1	0.954	−10.1 ± 0.1	0.492	−9.8 ± 0.2	0.335	−9.8 ± 0.5	0.886	−11.7 ± 0.1	0.712	−10.6 ± 0.1	0.305	−10.1 ± 0.2	0.398
**41**	−10.3 ± 0.1	0.322	−10.3 ± 0.1	0.258	−10.3 ± 0.2	0.307	−9.6 ± 0.1	0.793	−9.3 ± 0.1	0.780	−8.7 ± 0.1	0.242	−10.7 ± 0.1	0.747	−8.9 ± 0.2	0.222	−9.2 ± 0.1	0.352
**42**	−10.5 ± 0.1	0.302	−11.6 ± 0.1	0.328	−10.9 ± 0.1	0.324	−10.9 ± 0.1	0.315	−10.6 ± 0.1	0.303	−11.0 ± 0.1	0.258	−12.0 ± 0.2	0.683	−11.9 ± 0.1	0.264	−11.9 ± 0.1	0.241
**43**	−9.2 ± 0.1	0.221	−9.6 ± 0.2	0.324	−10.2 ± 0.1	0.301	−10.0 ± 0.1	0.310	−9.9 ± 0.2	0.283	−8.0 ± 0.1	0.296	−9.7 ± 0.1	0.665	−8.6 ± 0.1	0.226	−8.7 ± 0.1	0.275
**44**	−9.1 ± 0.3	0.255	−9.7 ± 0.2	0.249	−9.8 ± 0.2	0.289	−10.2 ± 0.2	0.239	−9.6 ± 0.1	0.243	−7.1 ± 0.1	0.278	−9.8 ± 0.2	0.915	−8.3 ± 0.1	0.172	−8.6 ± 0.1	0.239
**45**	−7.4 ± 0.2	0.246	−9.9 ± 0.4	0.236	−10.1 ± 0.3	0.234	−9.3 ± 0.2	0.303	−9.9 ± 0.2	0.287	−8.5 ± 0.2	0.411	−10.2 ± 0.2	0.904	−8.7 ± 0.1	0.142	−8.9 ± 0.1	0.253
**46**	−9.0 ± 0.2	0.286	−9.2 ± 0.1	0.908	−9.7 ± 0.1	0.264	−9.5 ± 0.2	0.295	−9.7 ± 0.2	0.290	−6.8 ± 0.1	0.738	−9.8 ± 0.2	0.943	−7.9 ± 0.1	0.292	−7.5 ± 0.2	0.262
**47**	−7.3 ± 0.1	0.267	−9.4 ± 0.2	0.300	−9.6 ± 0.1	0.295	−9.4 ± 0.1	0.282	−9.5 ± 0.2	0.225	−7.3 ± 0.1	0.261	−10.1 ± 0.2	0.891	−7.9 ± 0.1	0.254	−7.5 ± 0.1	0.330
**48**	−7.1 ± 0.2	0.274	−9.8 ± 0.3	0.298	−9.5 ± 0.1	0.405	−9.5 ± 0.2	0.245	−9.7 ± 0.1	0.229	−7.9 ± 0.1	0.264	−10.1 ± 0.1	0.912	−8.1 ± 0.1	0.187	−7.4 ± 0.1	0.179
**49**	−10.2 ± 0.1	0.582	−11.4 ± 0.1	0.329	−11.6 ± 0.1	0.513	−12.2 ± 0.2	0.319	−10.8 ± 0.2	0.671	−11.1 ± 0.6	0.512	−12.7 ± 0.2	0.878	−10.6 ± 0.1	0.352	−10.7 ± 0.1	0.308
**50**	−8.3 ± 0.1	0.281	−9.1 ± 0.1	0.515	−11.3 ± 0.2	0.712	−9.2 ± 0.2	0.846	−6.5 ± 0.2	0.428	−7.1 ± 0.3	0.258	−10.1 ± 0.1	0.910	−7.1 ± 0.1	0.240	−7.6 ± 0.1	0.488
**51**	−9.2 ± 0.1	0.510	−10.5 ± 0.5	0.459	−10.6 ± 0.2	0.808	−9.9 ± 0.2	0.643	−10.2 ± 0.1	0.372	−10.8 ± 0.1	0.726	−11.2 ± 0.2	0.978	−10.4 ± 0.2	0.568	−10.5 ± 0.2	0.525
**52**	−8.4 ± 0.1	0.494	−8.6 ± 0.2	0.334	−9.0 ± 0.1	0.352	−8.7 ± 0.2	0.336	−8.8 ± 0.2	0.793	−6.9 ± 0.2	0.297	−9.1 ± 0.3	0.916	−7.4 ± 0.1	0.256	−6.7 ± 0.1	0.293
**53**	−9.5 ± 0.1	0.358	−9.4 ± 0.1	0.375	−9.6 ± 0.1	0.556	−9.1 ± 0.2	0.337	−9.8 ± 0.1	0.653	−8.4 ± 0.1	0.389	−9.4 ± 0.1	0.889	−8.9 ± 0.1	0.281	−7.5 ± 0.2	0.368
**54**	−10.5 ± 0.1	0.365	−10.5 ± 0.1	0.312	−10.7 ± 0.1	0.348	−10.7 ± 0.1	0.354	−8.9 ± 0.1	0.307	−10.7 ± 0.6	0.324	−10.3 ± 0.2	0.453	−10.4 ± 0.1	0.242	−10.8 ± 0.1	0.372
**55**	−8.6 ± 0.1	0.376	−9.1 ± 0.1	0.361	−8.7 ± 0.2	0.297	−8.9 ± 0.1	0.374	−8.4 ± 0.2	0.338	−8.3 ± 0.1	0.364	−9.8 ± 0.1	0.998	−8.4 ± 0.1	0.374	−8.3 ± 0.2	0.412
**56**	−7.8 ± 0.1	0.256	−8.8 ± 0.2	0.252	−9.0 ± 0.1	0.307	−9.0 ± 0.1	0.300	−8.4 ± 0.2	0.641	−8.2 ± 0.1	0.257	−8.8 ± 0.3	0.856	−8.5 ± 0.1	0.193	−7.9 ± 0.2	0.250
**57**	−6.4 ± 0.1	0.275	−8.5 ± 0.4	0.436	−8.8 ± 0.2	0.281	−8.2 ± 0.4	0.232	−9.0 ± 0.1	0.260	−6.7 ± 0.1	0.240	−9.8 ± 0.2	0.643	−5.9 ± 0.1	0.172	−6.6 ± 0.2	0.228
**58**	−7.6 ± 0.1	0.247	−8.4 ± 0.1	0.471	−9.6 ± 0.1	0.230	−9.3 ± 0.1	0.246	−7.8 ± 0.2	0.508	−8.3 ± 0.1	0.173	−8.8 ± 0.2	0.995	−8.4 ± 0.1	0.186	−7.9 ± 0.2	0.249
**Natural Ligand d and Known Inhibitors *^e^***																		
**AA**	−8.6 ± 0.2	1.381	−8.4 ± 0.3	1.259	−7.8 ± 0.3	1.331	−7.5 ± 0.6	1.236	−8.1 ± 0.4	1.562	−7.7 ± 0.2	0.417	−8.2 ± 0.3	0.533	−7.8 ± 0.4	1.252	−7.7 ± 0.3	2.993
**CEL**	−10.7 ± 0.1	0.493	−11.6 ± 0.1	0.421	−10.9 ± 0.1	0.551	−10.8 ± 0.1	0.313	−10.9 ± 0.1	0.434	−11.0 ± 0.1	0.411	−12.7 ± 0.2	0.885	−11.1 ± 0.1	0.500	−11.4 ± 0.1	0.384
**DCL**	−8.7 ± 0.3	0.289	−8.2 ± 0.2	0.270	−8.3 ± 0.4	0.256	−8.1 ± 0.1	0.370	−8.7 ± 0.1	0.174	−8.5 ± 0.2	0.264	−8.6 ± 0.1	0.838	−8.0 ± 0.1	0.152	−7.9 ± 0.1	0.248
**IMD**	−8.2 ± 0.1	0.530	−10.3 ± 0.3	0.446	−10.7 ± 0.3	0.825	−10.5 ± 0.1	0.298	−9.2 ± 0.1	0.457	−9.5 ± 0.1	0.289	−9.9 ± 0.4	0.821	−9.6 ± 0.1	0.169	−9.1 ± 0.1	0.205
**NPX**	−8.6 ± 0.1	0.854	−8.5 ± 0.2	0.339	−8.7 ± 0.1	0.275	−8.4 ± 0.2	0.266	−8.3 ± 0.1	0.309	−9.3 ± 0.1	0.276	−8.6 ± 0.1	0.765	−8.7 ± 0.2	0.075	−8.8 ± 0.1	0.221
**SC58**	−10.7 ± 0.1	0.310	−12 ± 0.2	0.313	−10.9 ± 0.1	0.613	−10.6 ± 0.2	0.349	−11.2 ± 0.1	0.2631	−10.6 ± 0.1	0.449	−12.0 ± 0.2	0.786	−10.6 ± 0.1	0.268	−11.2 ± 0.1	0.356
***S*−IBP**	−7.5 ± 0.1	0.222	−7.6 ± 0.1	0.650	−8.2 ± 0.1	0.199	−8.2 ± 0.2	0.235	−8.5 ± 0.1	0.592	−8.2 ± 0.1	0.180	−7.5 ± 0.1	0.528	−7.7 ± 0.1	0.589	−7.8 ± 0.2	2.549
***R*−IBP**	−6.5 ± 0.1	0.377	−6.0 ± 0.1	1.065	−6.7 ± 0.1	0.550	−6.1 ± 0.1	1.376	−6.5 ± 0.2	0.267	−6.9 ± 0.1	1.434	−6.7 ± 0.1	0.665	−5.5 ± 0.1	0.574	−5.8 ± 0.1	0.751

*^a^***ES** = Enzyme Structure (**PDB** Code). *^b^* **VS** = Vina Scores (docking energies or affinities in kcal/mol) ± Standard Deviation (**SD**) of the affinities (best poses) after ten replicates per molecular docking calculation. *^c^* Root-mean-square deviation (**RMSD**) of atomic positions between the best poses after 10 replicates. *^d^* Arachidonic acid (**AA**). *^e^* Reported inhibitors: **CEL** = celecoxib (selective (S)). **DCL** = diclofenac (selective (S)). **IMD** = indomethacin (S). **NPX** = naproxen (N-S). **SC558** = 1-phenylsulfonamide-3-trifluoromethyl-5-parabromophenylpyrazole (S). **S-IBP** = (S)-ibuprofen (N-S). **R-IBP** = (R)-ibuprofen (non-inhibitor).

**Table A3:** Absorption, distribution, metabolism, and excretion–toxicity (ADMET) properties of best-docked compounds within the active site of COX-2.

Descriptors	11	29	42	49
MW	316.39	348.43	348.43	431.54
logP	2.0404	1.0172	0.9567	2.0612
logD	3.2	1.05	1.47	3.72
logSw	−2.82	−2.51	−2.41	−3.48
tPSA	36.92	68.15	79.15	69.92
RotatableB	4	2	3	5
RigidB	20	22	20	28
Flexibility	0.17	0.08	0.13	0.15
HBD	0	2	3	3
HBA	4	5	5	4
HBD_HBA	4	7	8	7
Rings	2	2	1	4
MaxSizeRing	9	14	17	9
NumCharges	0	0	0	0
TotalCharge	0	0	0	0
HeavyAtoms	23	25	25	32
CarbonAtoms	19	20	20	28
HeteroAtoms	4	5	5	4
ratioH/C	0.21	0.25	0.25	0.14
Lipinski_Violation	0	0	0	0
Sol (mg/L)	18,800.83	28,341.42	31,451.27	13,240.01
Sol Forecast Index	Good Solubility	Good Solubility	Good Solubility	Good Solubility
OB (VEBER)	Good	Good	Good	Good
OB (EGAN)	Good	Good	Good	Good
TrafficLights	0	0	0	1
Phospholipidosis	Non Inducer	Non Inducer	Non Inducer	Non Inducer
Overall Result	Accepted	Accepted	Accepted	Accepted

MW: Molecular weight in Da. log P = Octanol-water partition coefficient. tPSA = topological polar surface area. HBA = Hydrogen bond atoms. HBD = Hydrogen bond donors. Sol = solubility. OB = Oral bioavailability.
